# Vitamin C versus Cancer: Ascorbic Acid Radical and Impairment of Mitochondrial Respiration?

**DOI:** 10.1155/2020/1504048

**Published:** 2020-01-11

**Authors:** Rumiana Bakalova, Zhivko Zhelev, Thomas Miller, Ichio Aoki, Tatsuya Higashi

**Affiliations:** ^1^Department of Molecular Imaging and Theranostics, National Institute of Radiological Sciences (NIRS), National Institutes for Quantum and Radiological Science and Technology (QST), Chiba, Japan; ^2^Group of Quantum-State Controlled MRI, Institute for Quantum Life Science, National Institutes for Quantum and Radiological Science and Technology (QST), Chiba, Japan; ^3^Institute of Biophysics and Biomedical Engineering, Bulgarian Academy of Sciences, Sofia, Bulgaria; ^4^Medical Faculty, Trakia University, Stara Zagora, Bulgaria; ^5^IC-MedTech Co., San Diego, CA, USA

## Abstract

Vitamin C as a cancer therapy has a controversial history. Much of the controversy arises from the lack of predictive biomarkers for stratification of patients, as well as a clear understanding of the mechanism of action and its multiple targets underlying the anticancer effect. Our review expands the analysis of cancer vulnerabilities for high-dose vitamin C, based on several facts, illustrating the cytotoxic potential of the ascorbyl free radical (AFR) via impairment of mitochondrial respiration and the mechanisms of its elimination in mammals by the membrane-bound NADH:cytochrome b5 oxidoreductase 3 (Cyb5R3). This enzyme catalyzes rapid conversion of AFR to ascorbate, as well as reduction of other redox-active compounds, using NADH as an electron donor. We propose that vitamin C can function in “protective mode” or “destructive mode” affecting cellular homeostasis, depending on the intracellular “steady-state” concentration of AFR and differential expression/activity of Cyb5R3 in cancerous and normal cells. Thus, a specific anticancer effect can be achieved at high doses of vitamin C therapy. The review is intended for a wide audience of readers—from students to specialists in the field.

## 1. Introduction

In the April 2019 issue of *Nature Reviews Cancer*, Ngo et al. have described cancer vulnerabilities for high-dose vitamin C therapy [1]. The authors note that vitamin C as a cancer therapy has a controversial history, and much of the controversy arises from the lack of predictive biomarkers for stratification of patients, as well as a clear understanding of the mechanism of action and its multiple targets underlying the anticancer effect [2].

Our article links empirical and experimental observations from clinical practice that may, at first glance, seem disjointed. However, common characteristics of the pathologies discussed herein include mitochondrial dysfunction and severe inflammation. These are important targets for vitamin C and hallmarks of cancer. In particular, we expand the analysis of cancer vulnerabilities for high-dose vitamin C, based on several significant and indicative facts, illustrating the cytotoxic potential of the ascorbyl free radical (AFR) via impairment of mitochondrial respiration and the mechanisms of its elimination in mammals:

- AFR is an intermediate product from the oxidation of ascorbate by one-electron reduction of transition metal ions and/or interaction with free radicals [1]. AFR does not readily react with oxygen or other molecules to generate more reactive radicals and may temporarily accumulate in cells [3]
- AFR is a by-product of some vital biochemical reactions. Ascorbate participates in the recovery of hemoglobin from methemoglobin in erythrocytes, as well as in the enzymatic hydroxylation (e.g., during the synthesis of noradrenalin in the chromaffin granules of dopamine-synthesizing cells) [4–6]. Both processes are accompanied by AFR production
- NADH:cytochrome b5 oxidoreductase 3 (Cyb5R3) catalyzes rapid conversion of AFR to ascorbate, as well as reduction of other redox-active compounds, using NADH as an electron donor [7, 8]. Thus, Cyb5R3 eliminates AFR, restores the ascorbate “pool,” and helps maintain the NAD^+^/NADH ratio in cells. This enzyme is ubiquitously expressed in all mammalian cells, implying an important role in cell homeostasis
- Lack or downregulation of Cyb5R3 leads to development of recessive hereditary methemoglobinemia (RHM), accompanied by neuropathies and impairment of mitochondrial function [7, 9]. Ascorbate treatment has beneficial effects on methemoglobinemia, especially in patients with ascorbate deficiency. However, ascorbate deficiency does not explain the neuropathy and mitochondrial impairment associated with this pathology
- Downregulation of membrane-bound Cyb5R3 in cells leads to lower NAD^+^/NADH ratio, impaired mitochondrial respiration rate, and decreased ATP production, which is associated with higher sensitivity to oxidative stress [9]
- We propose that the accumulation of high concentrations of AFR in cells during oxidative stress and/or some specific metabolic activity is responsible for the impairment of mitochondrial respiration in the absence or downregulation of Cyb5R3, particularly the enzyme located on the outer mitochondrial membrane (OMM Cyb5R3). This can induce cytotoxicity in mammalian cells. Perhaps this is why enzymes found in plants that convert ascorbate to AFR (ascorbate oxidase and ascorbate peroxidase) did not evolve in animals and humans [3]

*Is AFR harmful for mitochondria and can pharmacological doses of vitamin C attack cancer cells by inhibiting membrane-bound Cyb5R3 and impairing mitochondrial respiration?*

## 2. Cyb5R3/VDAC1 Is a Redox-Cycling System That Converts AFR to Ascorbate

The discovery of enzyme activity of Cyb5R3 dates back to 1951, when Lehninger reported that isolated rat liver mitochondria catalyze rapid oxidation of NADH in the presence of added cytochrome c. This mitochondrial NADH:cytochrome c oxidoreductase activity was found to be independent of the respiratory chain. Thirty years later, it was found that this enzyme is NADH:semidehydroascorbic acid reductase, which uses a cytochrome b5-like hemoprotein of the outer mitochondrial membrane (OMM cytochrome b5), which is distinct from microsomal cytochrome b5 [10]. A similar enzyme was also found in *Saccharomyces cerevisiae*—mitochondrial NADH:cytochrome b5 reductase, which catalyzes the reduction of D-erythroascorbic acid radical [11].

Recently, OMM NADH:cytochrome b5 reductase was classified as OMM Cyb5R3 (NADH:ferricytochrome b5 oxidoreductase 3; EC 1.6.2.2.). *Cyb5R3 belongs to the class of cancer-related and disease-related genes. Cyb5R3 enzyme belongs to the class of potential drug targets and predicted membrane proteins (according to The Human Protein Atlas)*. The Cyb5R3 protein is encoded in two isoforms: (i) soluble, exclusively expressed in erythrocytes, and (ii) membrane-bound, expressed in all other cells and anchored to the outer mitochondrial membrane, endoplasmic reticulum, and plasmatic membrane [8]. Deficiency of cytosolic Cyb5R3 causes type I RHM, which is a benign condition with mild cyanosis, fatigue, and shortness of breath upon exertion [9, 12]. Deficiency of membrane-bound Cyb5R3 causes incurable type II RHM, accompanied by severe neuropathy, permanent cyanosis, and impairment of mitochondrial respiration [9, 12].

OMM Cyb5R3 is functionally connected to the voltage-dependent anion channel 1 (VDAC1), the most abundant protein of the outer mitochondrial membrane [8]. Initially, it was believed that VDAC1 possesses NADH-dependent oxidoreductase activity. However, VDAC1 does not contain flavin, heme, and/or metal ions in its active center, nor any other catalytic motif of the known NAD(P)H-dependent oxidoreductases. Recombinant VDAC1 and VDAC1 purified from comigrating proteins either lack oxidoreductase activity or exhibit very low activity [8, 13, 14]. The purity of VDAC1 preparations studied usually reaches only 90-95%. In all cases, other proteins of equal molecular weights (32-35 kDa) are present, which affect the efficiency of separation [15–18]. It was reported that Cyb5R3 consists of 5-10% of the purified VDAC1 [15, 18]. The oxidoreductase activity ascribing to VDAC1 is inhibited by nonspecific VDAC blockers [15, 16, 19], as well as by specific Cyb5R3 inhibitors [19]. This is direct evidence that oxidoreductase activity of VDAC1 is due to the presence of Cyb5R3 in the samples. It seems that both proteins cofunction and could be considered as one “redox-cycling system” of the outer mitochondrial membrane. Recent studies on cells treated with paraquat (considered to be carcinogenic) indicate that VDAC1 maintains membrane integrity and keeps mitochondria intact, while Cyb5R3 acts as an oxidoreductase [8, 20].

## 3. The Cyb5R3/VDAC1 Redox-Cycling System Reduces AFR and Helps Maintain Mitochondrial Homeostasis

The crucial role of Cyb5R3 in regeneration of AFR to ascorbate and mitochondrial homeostasis has been demonstrated by Shirabe et al. on human dermal fibroblasts (HDFs), derived from a patient with type II RHM with a point mutation in the Cyb5R3 gene [21]. This mutation results in a complete absence of membrane-bound Cyb5R3 in the cells. The authors have reported severely decreased cellular NADH:semidehydroascorbic acid reductase activity and rotenone-insensitive NADH:cytochrome c reductase activity, compared to neonatal HDFs. These data suggest that OMM Cyb5R3 and its NADH:semidehydroascorbic acid reductase activity might contribute to the protection and normal functioning of the cell, as well as protection of its mitochondrial electron transport chain (ETC).

Siendones et al. have found that membrane-bound Cyb5R3-deficient HDFs, derived from two patients with similar mutations, are characterized by the decrease of several parameters: NAD^+^/NADH ratio, mitochondrial respiration rate, activities of mitochondrial Complexes I-IV, and ATP production [22]. This is associated with higher sensitivity of Cyb5R3-deficient HDFs to oxidative stress and accelerated senescence, compared to neonatal HDFs. The role of Cyb5R3 in the modulation of mitochondrial function has also been evaluated in Cyb5R3-siRNA-silenced MRC-5 normal fibroblasts [22]. Downregulation of Cyb5R3 is accompanied by a significant decrease of the NAD^+^/NADH ratio and mitochondrial respiration rate. Cyb5R3-siRNA-silenced MRC-5 cells and type II RHM HDFs displayed low proliferation rates and increased cell death. This is consistent with impairment of mitochondrial metabolism and reduction of oxidative phosphorylation, based on analysis of oxygen consumption. The authors suggest that impairment of Cyb5R3 expression and/or its AFR reductase activity may result not only in NADH accumulation but also in defects of mitochondrial ETC due to increased oxidative damage, ultimately leading to senescence.

Recently, it was reported that Cyb5R3 is essential for cardiomyocyte function [23]. Cardiomyocyte-specific inducible Cyb5R3-knockout mice (Myh6-Cre^ERT2^-flox/flox) show a loss of mitochondrial biogenesis, associated with 30% loss of total ATP, 50% loss of Complex IV activity, and 25% loss of Complex IV protein quantity [23]. RNA_seq_ pathway analysis indicated significantly decreased expression of Complexes I, III, and IV after downregulation of membrane-bound Cyb5R3 in cardiomyocytes. This study shows that Cyb5R3 is essential for normal mitochondrial function and cell survival.

The studies described above suggest that the presence of OMM Cyb5R3 is essential for the following parameters at normal conditions: activity of mitochondrial ETC, oxygen consumption, ATP production, and resistance to oxidative stress.

*In vitro* studies on isolated mitochondria indicate that the OMM Cyb5R3/VDAC1 complex is responsible for the transfer of electrons from cytosolic NADH into mitochondria and that the process is dependent to Complex IV [24, 25]. This is accompanied by oxygen uptake, proton pumping, and generation of mitochondrial potential, supported by small catalytic amounts of external (mitochondrial) cytochrome c. Their data also demonstrate that cytochrome c-dependent NADH oxidation is strongly inhibited by dextran sulfate (500 kDa)—an inhibitor of VDAC1. They have suggested that in physiological conditions, cytochrome c might be transferred in a very limited amount outside the mitochondria so as to promote the activation of the Cyb5R3-dependent electron transport pathway. The process is highly dependent on induction of contact sites in the outer and inner mitochondrial membranes (membrane remodeling), as well as on inhibitors of VDAC1. The activity of this Cyb5R3/VDAC1-dependent system becomes functional in removing the excess cytosolic NADH and is essential for cell survival under impairment of the ETC at the level of one of the first respiratory complexes. Since the Cyb5R3-catalyzed electron transfer generates an electrochemical membrane potential associated with the activity of the cytochrome oxidase [24, 26, 27], it represents an additional pathway for providing energy to cells. This aspect is essential for the cells in which oxidative phosphorylation activity is not properly supported by the respiratory chain.

The same authors also demonstrate a nonenzymatic induction of this alternative pathway for ATP synthesis in mitochondria [24, 25]. They added ascorbate (instead of NADH) and small catalytic amounts of external cytochrome c to intact mitochondria and detected oxygen uptake, accompanied by cytochrome c reduction and ascorbate oxidation [24, 25]. These processes are dependent on Complex IV and the induction of contact sites in mitochondrial membrane and are sensitive to inhibitors of VDAC1. It can be assumed that oxidation of ascorbate by small catalytic amounts of external cytochrome c leads also to AFR production. AFR may enter the mitochondria via VDAC1 and affect mitochondrial respiration at Complexes III-IV—a hypothesis, which is described below in the context of the anticancer effect of high doses of vitamin C.

## 4. The Cyb5R3/VDAC1 Redox-Cycling System in Cancer

Studies discussed above suggest that OMM Cyb5R3/VDAC1 is vital for mitochondrial homeostasis, protection against oxidative stress, prevention of cell senescence, and cellular longevity. These events clearly relate to AFR elimination and maintenance of the cytosolic NAD^+^/NADH ratio—crucial factors for cell survival.

It has been shown that VDAC1 is highly expressed in all cells as a consequence of exposure to various toxic substances and plays a crucial role in the protection against intoxication [8, 28, 29]. VDAC1 interacts with both proapoptotic and antiapoptotic factors, which makes it a gatekeeper for mitochondria-mediated cell death or survival signaling pathways [30, 31]. This illustrates the complexity of VDAC functions in normal and cancer cells. It has been observed that overexpression of VDAC1 in cancer cells is associated with high metastatic potential, low therapeutic efficiency, and poor prognosis [30, 31]. In this case, VDAC1 appears to be involved in protecting mitochondria from reactive oxygen species (ROS), functioning as a prosurvival pathway [28–30].

Cyb5R3 is also recognized as a carcinogen detoxification gene [32, 33], although its role in carcinogenesis is not yet well studied. Single reports on the role of OMM Cyb5R3 in cancer have appeared over the past 10 years [32, 34, 35]. It was found that the enzyme is overexpressed in cancer cells, protecting them against oxidative stress and induction of apoptosis [32, 34, 35].

Rajcevic et al. have analyzed the proteomic profile of metabolic proteins in the invasive glioblastoma phenotype by applying a functional analysis, using isobaric peptide tagging chemistry (iTRAQ) combined with bioinformatics analysis (Ingenuity® Pathway Analysis) [35]. Cyb5R3 is identified as a key protein in a canonical pathway of the cancerous phenotype associated with mitochondrial dysfunction.

Two groups have reported that membrane-bound Cyb5R3 overexpression and polymorphism are associated with breast cancer risk in women [32, 34]. Blanke et al. have found that one variant of Cyb5R3 is significantly overexpressed in African American women with breast cancer, which is accompanied by impaired detoxification of aromatic and heterocyclic amine mammary carcinogens in cigarette smoke [32]. The authors suggest that overexpression of membrane-bound Cyb5R3 should be considered as a valuable marker in etiologic studies of prostate, bladder, and colon cancers, which are also associated with exposure to arylamine and heterocyclic amine carcinogens.

A large cohort study on patients with estrogen receptor-negative (ER^neg^) and progesterone receptor-negative (PR^neg^) breast tumors reveals a central role of membrane-bound Cyb5R3 in extravasation/colonization of cancer cells and metastasis formation at distinct sites (e.g., lung) [34]. Immunohistochemical analysis has identified a significant correlation between high Cyb5R3 expression and poor disease-free (DFS; *p* = 0.02) and overall survival (OS: *p* = 0.04). Multivariate analysis revealed about 2-fold higher risk of poor outcome for ER^neg^/PR^neg^ patients exhibiting strong staining of Cyb5R3, compared to those expressing weak staining. Cyb5R3 gene knockdown using siRNA in metastatic cells leads to a significantly decreased tumor burden in lungs when injected intravenously in immunodeficient mice. The cellular effects of Cyb5R3-siRNA knockdown showed signaling alterations associated with extravasation, such as transforming growth factor beta (TGF*β*) and hypoxia-inducible factor alpha (HIF*α*) pathways, and strong suppression of proliferation. It is interesting to note that Cyb5R3-siRNA-silenced cancer cells stop proliferation and metastasis, but they continue to survive [34].

## 5. AFR in Mitochondrial Homeostasis at Low and High Doses of Vitamin C

Our hypothesis is focused on the intracellular generation of AFR at low and high doses of vitamin C, together with its effect on mitochondrial respiration, intracellular redox balance, and energy metabolism mediated by the OMM Cyb5R3/VDAC1.

*We propose that vitamin C can function in “protective mode” or “destructive mode” affecting cellular homeostasis, depending on the OMM Cyb5R3 expression/activity and intracellular “steady-state” concentration of AFR. The hypothesis is focused on the effects of AFR in the mitochondrial intermembrane space and the cytosol*.

Membrane-bound Cyb5R3 has several characteristics that could be crucial to mitochondrial homeostasis:

- The redox potential of the OMM-bound cytochrome b5 varies from -160 mV to -272 mV, depending on the NAD^+^/NADH ratio [8, 13]. Therefore, OMM Cyb5R3 can catalyze the reduction of a variety of redox cyclers in addition to AFR, such as quinone-like compounds, bioreductive xenobiotics/drug, and nitrocompounds [8, 17]. These redox cyclers can influence the intracellular redox state
- Membrane-bound Cyb5R3 helps maintain the cytosolic NAD^+^/NADH ratio, supplying cells with reducing equivalents by transferring electrons from cytosolic NADH to the respective membrane components (electron acceptors and carriers) [7, 8, 10, 11, 22]
- The membrane-bound Cyb5R3 is considered to be one of the major enzymes that maintain the NAD^+^/NADH ratio, by using the coenzyme Q (CoQ) “pools” of the outer mitochondrial membrane and endoplasmic reticulum [8, 10, 11]

CoQ is most likely the main acceptor of excess electrons, as it is the most abundant membrane-bound redox-active compound in the cell. CoQ is also a key electron carrier in mitochondria. Therefore, outside the inner mitochondrial membrane, CoQ can serve as a “buffer” for excess-reducing equivalents, accepting electrons from excess cytosolic NADH via Cyb5R3.

We also assume that the ascorbate could serve as a “buffer” of excess-reducing equivalents in the intracellular aqueous phase of cancer cells due to their oxidative environment, because ascorbate is one of the most abundant cytosolic redox-active compounds. Steady-state levels of ascorbate are maintained by Cyb5R3 [6–8], and they are significantly higher in cancer cells compared to normal cells [1, 36].

**Figure 1:**
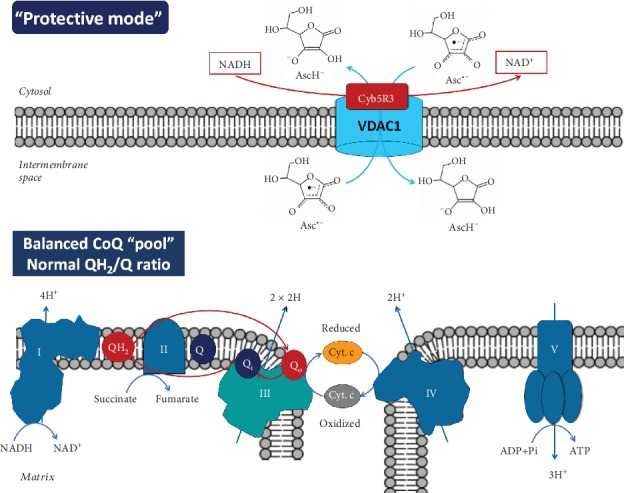
“Protective mode” of action of the OMM Cyb5R3/VDAC1 at low (steady-state) concentrations of vitamin C in normal cells (at normoxia). OMM Cyb5R3/VDAC1 converts ascorbyl free radical (AFR; Asc^·-^) to ascorbate (AscH^−^), using cytosolic NADH as an electron donor. This prevents effects on mitochondrial respiration and all complexes operate in a “normal mode,” creating a proton gradient and leading to normal ATP synthesis. The CoQ “pool” is balanced [39], and the Qo site of Complex III transfers one electron to cytochrome c and the second electron to ubiquinone in the Qi site. VDAC1: voltage-dependent anion channel 1; Cyb5R3: NADH:cytochrome b5 oxidoreductase 3; OMM: outer mitochondrial membrane; Cyt. c: cytochrome c.

**Figure 2:**
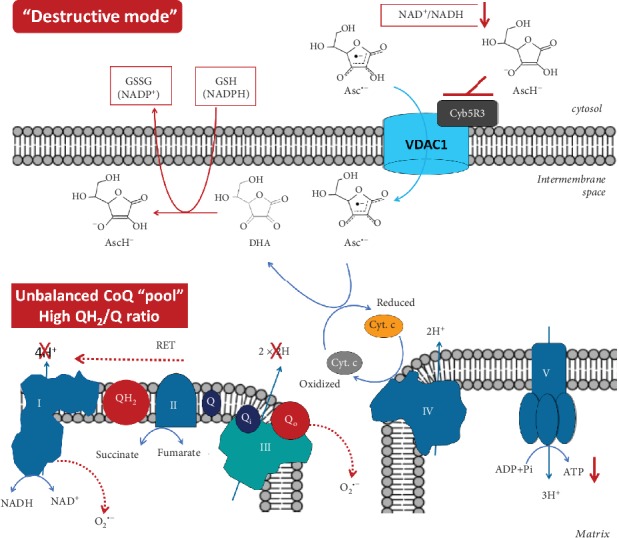
“Destructive mode” of action of ascorbyl free radical via the OMM Cyb5R3/VDAC1 at high (“therapeutic”) concentrations of vitamin C in cancer cells (at normoxia). High intracellular concentration of ascorbate may induce Cyb5R3 end-product inhibition, accompanied by elevated levels of ascorbyl free radical (AFR) in the mitochondrial intermembrane space and decrease of the NAD^+^/NADH ratio in the cytosol. AFR may transfer one electron to oxidized cytochrome c, causing a partial (or complete) arrest of electron flow between Complex III and Complex IV. Rapid reduction of cytochrome c by AFR effectively competes with and perturbs the proton pumping at Complex III, as well as the temporal electron transport provided by CoQ cycles of Complex III. When Complex III is blocked, succinate (from the citric acid cycle) builds up, mitochondrial membrane potential rises, and the CoQ “pool” becomes unbalanced. Reverse electron transport (RET) to Complex I is driven, which causes a superoxide “burst” [95, 96]. RET is also accompanied by synthesis of succinate and NADH. We propose that the “destructive mode” caused by high-dose vitamin C may contribute to RET not only during tissue reperfusion at angiogenesis but also during tumor hypoxia and normoxia in cancer cells. In addition, the reduction of cytochrome c by AFR results in production of DHA. DHA is converted to ascorbate by glutathione, which provokes a depletion of reducing equivalents in cancer cells—a crucial factor for their survival. VDAC1: voltage-dependent anion channel 1; Cyb5R3: NADH:cytochrome b5 oxidoreductase 3; OMM: outer mitochondrial membrane; Cyt. c: cytochrome c; RET: reverse electron transport; DHA: dehydroascorbate; AscH^−^: ascorbate in anion form; Asc^·-^: ascorbyl free radical.

*Figures 1 and 2 represent two modes of action of vitamin C on mitochondrial homeostasis via generation of AFR and its elimination by the OMM Cyb5R3/VDAC1: (i) “protective mode” at low/normal (steady-state) doses and (ii) “destructive mode” at high doses of vitamin C*.

Ascorbate and AFR exist as anions, which imply their penetration from the cytosol into the mitochondrial intermembrane space and back through the VDAC1 (Figure 1).

In normal cells (Figure 1), low concentrations of AFR should be generated due to the low (steady-state) intracellular levels of vitamin C, as a result of (i) relatively low expression of vitamin C transport proteins [37] and (ii) low steady-state levels of ROS and normal levels of reducing equivalents [38]. Both factors are responsible for the limited production of AFR, which is a product of one-electron oxidation of ascorbate. Since AFR is relatively stable, it could be rapidly eliminated by the OMM Cyb5R3/VDAC1. This prevents effects on mitochondrial respiration, and all complexes will operate in a “normal mode.” Electrons are transferred from Complex III's Qo site of the CoQ “pool” to cytochrome c, creating a proton gradient and leading to normal ATP synthesis. In this case, the CoQ “pool” is balanced and the Qo site of Complex III transfers one electron to cytochrome c and the second electron to ubiquinone while bound in the Qi site [39]. This model is typical for normal (noncancerous) cells.

In cancer cells, relatively high “steady-state” levels of vitamin C can be achieved due to overexpression of its transport proteins [1, 37, 40]. Cancer cells are also characterized by permanent oxidative stress and redox imbalance, accompanied mainly by overproduction of superoxide, hypoxic environment, and overproduction of cytosolic NADH due to upregulation of the aldehyde dehydrogenase pathway [41, 42]. These distinct characteristics suggest increased production of intracellular AFR at “steady-state” levels of vitamin C in cancer cells, compared to normal cells. On the other hand, AFR should be rapidly eliminated in the mitochondrial environment of cancer cells due to overexpressed Cyb5R3/VDAC1 [30, 32, 34, 35] and high cytosolic levels of NADH [38, 40, 41] as an electron donor. Overexpression of the Cyb5R3/VDAC1 appears to be a compensatory mechanism that protects cancer cells and their mitochondria by regulating AFR accumulation at “steady-state” conditions, while also maintaining the cytosolic NAD^+^/NADH ratio.

## 6. What Processes Can Be Induced in Cancerous Mitochondria at High “Therapeutic” Intracellular Concentrations of Vitamin C?

As well described by Ngo et al. and others, the first consequence of the high-dose “therapeutic” vitamin C is to selectively elevate its concentration in cancer cells [1, 36, 43]. Chen et al. have reported that vitamin C in pharmacological doses selectively generates AFR and hydrogen peroxides in the extracellular fluids of cancer-bearing patients [44]. Keshari et al. have studied ascorbate/dehydroascorbate ratios in solid tumors in vivo as a marker of oxidative stress at different expressions of glucose transporters [45, 46]. The authors have detected AFR as an intermediate product, using time-resolved MR spectroscopy. These studies show that AFR is also generated inside the cells. It should be clarified that high intracellular levels of AFR can only be achieved with intravenous vitamin C administration, because ascorbate concentrations in the plasma and tissues are tightly controlled as a function of the oral dose [47].

We suppose that high “therapeutic” intracellular concentration of ascorbate in cancer cells is then likely to induce “enzyme (Cyb5R3) end-product inhibition”—a negative feedback, regulating any enzymatic pathway (Figure 2). The inhibition of OMM Cyb5R3 will result in the accumulation of high amounts of AFR under the permanent oxidative stress within these cells.

In the mitochondrial intermembrane space, AFR may transfer one electron to cytochrome c, causing a partial (or complete) arrest of electron flow between Complex III and Complex IV. It has been shown that the rate of reduction of cytochrome c by AFR, with production of DHA, is orders of magnitude faster than the reduction of cytochrome c by ascorbate [48, 49].

Rapid reduction of cytochrome c by AFR effectively competes with the electron transport from the CoQ cycle of Complex III to the cytochrome c, which perturbs the proton pumping. Competitive inhibition by AFR, bypassing Complex III to cytochrome c, drives the CoQ “pool” to a more reduced (overcharged) state. If this overcharging phenomenon occurs, there will be insufficient ubiquinone, available for binding at the Qi site. This unbalances the Qo/Qi cycle. It also increases the lifetime of semiubiquinone in the Qo pocket, allowing oxygen to accept the second electron from ubiquinol during the second step of the CoQ cycle, yielding superoxide (Figure 2) [50].

Briefly, we propose that a high level of AFR in the intermembrane space of mitochondria blocks Complex III, at least partially, via unbalancing the CoQ “pool” and impairing mitochondrial respiration in vitamin C-treated cancer cells. Precisely timed redox sequences responsible for the efficient flow of electrons from Complex III to oxidized cytochrome c are vulnerable to AFR flux. This drives the CoQ “pool” to a more reduced (overcharged) form. This also causes the lifetime of semiubiquinone in the Qo pocket to lengthen, allowing oxygen to accept the second electron from ubiquinol during the second step of the CoQ cycle, yielding superoxide. This can interfere with the timing of the Qo/Qi cycle and consequently the CoQ “pool” redox balance, impairing mitochondrial respiration. A highly reduced CoQ “pool” inhibits proton pumping, yielding up to 80% lower ATP plus higher levels of superoxide. We further propose that the damaging effect of high-dose vitamin C on cancer cells is similar to the mechanism of ischemia-reperfusion injury (IRI) or reoxygenation damage [51]. In IRI, the mitochondrial CoQ “pool” becomes highly reduced by stopped flow through Complex III.

It is interesting to note that this mechanism can influence the mitochondrial ATP production in different directions depending on the cell type. In the case of cancer cells, this mechanism should result in a significant reduction of mitochondrial ATP and increase of superoxide production (Figure 2).

It has been generally accepted that cancer cells use glycolysis as a source of ATP (Warburg effect) and do not rely on mitochondrial respiration for ATP production. However, recent studies demonstrate that the majority of ATP in cancer cells is produced by mitochondria and some tumors show heavy dependence on oxidative phosphorylation [52–56]. Many solid tumors are poorly perfused and have a limited supply of glucose, but enough oxygen to generate mitochondrial ATP [57]. The ETC is able to function optimally at oxygen levels as low as 0.5% [58]. Therefore, blocking mitochondrial ATP production will induce cell death in poorly perfused tumors.

Even ignoring the dependence of cancer cells on mitochondrial ATP, a function of the Krebs cycle (at Complex II) is necessary for the supply of metabolites for synthesis of nucleic acids, fatty acids, etc. Blocking the ETC will stop this supply and suppress proliferation. Krebs cycle metabolites (such as succinate, fumarate, and itaconate) are also coupled with nonmetabolic signaling in cancer and immune cells, which is crucial for cancer progression and invasion [59, 60].

The key consideration in targeting mitochondria by high-dose vitamin C (via AFR) is that normal cells rely mostly on mitochondrial respiration for ATP production. As was mentioned above, high concentrations of AFR cannot be reached in normal cells, especially in vivo in the absence of oxidative stress, due to lower expression of glucose/ascorbate transport proteins [36, 47] and low amounts and normal functioning of Cyb5R3 [32, 33]. However, normal cells should also be vulnerable to high concentrations of AFR generated under oxidative stress. Perhaps, this is a part of the dual effect of vitamin C, which can act as an antioxidant or prooxidant, depending on the environment [61].

It is shown that in the case of noncancer cells with mitochondrial deficiency, high-dose vitamin C could even increase ATP production. Eleff et al. reported that administration of vitamin C and quinone-like provitamin menadione on the skeletal muscles of a 17-year-old patient with a severe defect in Complex III of the mitochondrial ETC increased the recovery rate, compared to the recovery rate of the young female controls [62]. This rare defect included a stop-codon mutation (G15242A) in the mtDNA-encoded cytochrome b gene, which was accompanied by an effective prevention of aerobic metabolism and oxidative phosphorylation. The authors suppose that both substances bypass the deficient Complex III as electron transfer mediators to carry the electrons from CoQ to cytochrome c. Thus, they increased ATP production from mitochondria compared to the initial level. It is interesting to note that redox cycles of vitamin C and menadione are mediated by Cyb5R3/VDAC1 [8, 17] and the enzyme activity could be important for this bypass. In this patient, the combination of vitamin C and menadione resulted in the production of more ATP than in the case of vitamin C applied alone. The authors suggest that both substances can directly reduce cytochrome c, since the reduction potential of cytochrome c is over +200 mV—more positive than those of ascorbate and menadione [62–65].

## 7. What Could Be the Consequences of AFR-Mediated Electron Transfer in Cancerous Mitochondria from High-Dose Vitamin C Therapy?

- Induction of reverse electron transport in the respiratory chain and acceleration of superoxide production at Complex I, as well as Complex III (Figure 2) [50, 65–67]. Degradation of Complex I when the CoQ “pool” is unbalanced, and Complex III is dysfunctional [67]. In hypoxic conditions (common to many types of cancer cells), these redox reactions occur even faster, exponentially increasing superoxide production [50, 65–67].
- Superoxide-mediated destruction of Fe/S clusters from the complexes and induction of Fenton's reactions in mitochondria [68–70], in the presence of AFR and/or ascorbate
- Mitochondria within cancer cells are vulnerable to a “destructive mode” of action from high-dose vitamin C, which leads to their collapse

In addition, the conversion of AFR to DHA and subsequent reduction of DHA to ascorbate by glutathione- and NADPH-dependent pathways will promote a depletion of glutathione and reducing equivalents in the cancer cells leading to severe oxidative stress—crucial factors for induction of apoptosis and cell death [38].

An important consequence of the reduction of cytochrome c by AFR is the transfer of electrons from cytoplasmic NADH to Complex IV, which is accompanied by production of 1 mol ATP per 1 mol NADH—suggesting the likelihood of thermogenesis [71, 72]. Cancer cells often express heat shock proteins and are very sensitive to the elevated temperature of their environment [38, 73]. This may also contribute to converting “cold” tumors into “hot” tumors, an important cell death-facilitating factor potentially from combining high-dose vitamin C therapy with applications in immunotherapy [74].

It is also reported that NADH reduces conduction of VDAC by partial channel block and/or modulation of its activity [75, 76]. As ATP and many other vital substances pass from the mitochondria into the cytosol through VDAC [77, 78], partial inhibition of the VDAC channel would also contribute to the impairment of mitochondrial respiration and decrease of cell viability.

Recent studies show that Cyb5R3/VDAC1 can activate bioreductive antitumor drugs, which in combination with vitamin C may affect mitochondrial activity, exhibiting additive or synergistic anticancer effects [17, 79, 80]. For example, vitamin C can sensitize cancer cells to redox-responsive chemotherapeutics (e.g., furanonaphtoquinones) after their bioreductive activation via Cyb5R3/VDAC1 [81, 82]. In this case, the “therapeutic” intravenous dose of vitamin C could be decreased to more tolerant values. It has been reported that high doses of vitamin C exhibit selective antitumor effects in combination with menadione—an analogue of 1,4-naphoquinone [83–85]. A recent study demonstrates that high/tolerable concentrations of menadione inhibit Cyb5R3 [86]. It can be assumed that menadione-mediated inhibition of Cyb5R3 is involved in potentiation of the anticancer effect of high-dose vitamin C via overproduction of AFR and subsequent impairment of mitochondrial respiration (as we assume with our “model”). Moreover, vitamin C/menadione could achieve a selective cytotoxicity against cancer cells due to the rapid UBIAD1-mediated conversion of menadione to vitamin K2 in normal cells and downregulation of UBIAD1 in the majority of cancers leading to strong inhibition of this conversion [87, 88]. These assumptions could be a prerequisite/basis for the design of future studies aimed to elucidate the molecular mechanisms of the synergistic anticancer effects of vitamin C and vitamin C/menadione with conventional chemotherapeutics, described in the literature [89]. Clarifying the effects of conventional anticancer drugs and high-dose vitamin C on Cyb5R3 may also be a prerequisite for predicting the effectiveness of vitamin C in adjuvant settings and stratification of patients with good/poor prognosis.

## 8. Concluding Remarks and Outstanding Questions

The high concentrations of cytochrome c in mitochondria [90], as well as the possible existence of dedicated pools of CoQ and cytochrome c (as supported by the “plasticity model” of mitochondria) [90, 91], could limit the proposed direct effects of AFR on oxidative phosphorylation, as described in Figure 2. In fact, reported concentrations of vitamin C in cells under normal conditions are significantly higher than those of cytochrome c (~10-30 nmol/mg protein versus ~0.1-1 nmol/mg protein) [90, 92]. In this case, ascorbate and AFR concentrations in cancer cells after treatment with high doses of vitamin C should be much higher than cytochrome c concentrations [93]. This supports the possibility of direct electron transfer from AFR to cytochrome c at Complex IV. Remodeling of the mitochondrial membrane and, in particular, the contact sites of Cyb5R3/VDAC1/Complex IV to optimize performance can explain the different efficiency and selectivity of high-dose vitamin C therapy when combining with different anticancer drugs (depending on their mechanisms). In addition, the abovementioned consequences of AFR-mediated electron transfer in cancerous mitochondria at high-dose vitamin C therapy may explain the induction of apoptosis even without strong inhibition of oxidative phosphorylation and ATP production. The mechanism, described in Figure 2, may not cause strong cancer cell death, but it can damage the mitochondria, stop proliferation, and keep cancer cells latent, so they become vulnerable to the immune system of the intact organism.

The analysis of the literature raises several outstanding questions that need experimental verification:

1. Does AFR impair mitochondrial respiration by reducing cytochrome c, causing an arrest of electron flow between Complexes III and IV, a reverse electron transport, and an induction of oxidative stress in the absence or downregulation of Cyb5R3/VDAC1
2. Does vitamin C in low/normal doses work in “protective mode” in normal and cancer cells? If yes, is this related to maintaining the balance of the CoQ “pool” and cytosolic reducing equivalents
3. Does vitamin C in high doses work in “destructive mode” in cancer cells, but not in normal cells? If yes, is this related to maintaining the imbalance of the CoQ “pool” and cytosolic reducing equivalents
4. What are the levels of mitochondrial superoxide, succinate, and ATP in normal and cancer cells of the same origin, treated with low, moderate, and high doses of vitamin C? Is there a difference in the levels of mitochondrial superoxide, succinate, and ATP in cells with different proliferative index, treated with vitamin C
5. Does the effect of pharmacological vitamin C relate to the expression and activity of membrane-bound Cyb5R3/VDAC1? Can high doses of vitamin C attack cancer cells only by inhibiting Cyb5R3/VDAC1 and specific destruction of cancer mitochondria
6. What is the impact/significance of Cyb5R3/VDAC1 as a predictive biomarker for stratification of cancer-bearing patients for high-dose vitamin C therapy
7. Does pharmacological vitamin C have a potential as adjuvant cancer therapy in combination with redox-responsive drugs

Our article is intended to provoke the design of new experiments (in vitro and in vivo) that can provide evidence Pro and Con for the assumptions made, to change the “status quo” in the controversial field of “pharmacological vitamin C.”

If vitamin C can clinically exploit the pathway presented here, we suggest it offers a new, exciting rationale for cancer studies. Perhaps this orthogonal mechanism of action can safely and more effectively compliment current cancer treatments in the adjuvant setting and help to let the phoenix fly [94].

## Abbreviations

AFR: Ascorbyl free radical
CoQ: Coenzyme Q
Cyb5R3: NADH:cytochrome b5 oxidoreductase 3
DHA: Dehydroascorbic acid
HDF: Human dermal fibroblast
OMM: Outer mitochondrial membrane
RHM: Recessive hereditary methemoglobinemia
VDAC1: Voltage-dependent anion channel 1.
